# Editorial: The MACC1 network in cancer

**DOI:** 10.3389/fonc.2023.1343812

**Published:** 2024-07-05

**Authors:** Wolfgang Walther, Franziska Siegel, Ulrike Stein

**Affiliations:** ^1^ Experimental and Clinical Research Center, Department of Translational Oncology of Solid Tumors, Charité - Universitätsmedizin Berlin and Max-Delbrück-Center for Molecular Medicine in the Helmholtz association, Berlin, Germany; ^2^ Research & Early Development Oncology, Bayer AG, Berlin, Germany

**Keywords:** metastasis, MACC1, prognosis, prediction, intervention, solid cancer

The novel, previously undescribed gene metastasis-associated in colon cancer 1 (MACC1) (1) was initially published in 2009. Since then, more than 330 succession papers (PubMed) from research groups worldwide were published until today; including meta-analyses of solid cancers, hepatocellular, gastrointestinal cancer, CRC, gastric, gynecological and breast cancer. Thereby, the prognostic and predictive value of MACC1 for tumor initiation, progression and metastasis in the whole spectrum of solid cancers was strongly confirmed. MACC1 has been established by many groups as key player, prognostic and predictive biomarker for tumor progression and metastasis in more than 20 solid cancers. MACC1 levels in the primary tumors or patient blood were significantly higher in those cancers that metachronously developed distant metastases compared to those, which did not metastasize. Based on the RNA or protein expression levels of MACC1 in patient tumor or blood, a clear prognosis can be made whether a patient develops metastasis (or not), linked to shorter survival. This prognostication can be improved when combining markers of MACC1 networks or axes. Thus, the thorough analysis of key drivers for metastasis such as MACC1, their regulation, role in cancer cell signaling, functional impact for metastasis, and on novel intervention strategies to restrict metastasis of solid cancers is of overarching interest. Taken together, MACC1 has been established as strong metastasis biomarker in tumor tissues and liquid biopsies for clinical disease prognosis and prediction of therapy response in cancer patients.

Despite the improvements made for solid cancer treatments, metastasis still represents a major treatment challenge, which is critically limiting successful therapy in many cancer types. Cancer metastasis is the most lethal attribute of cancer, since it is directly linked to patient survival (patient survival is about 80% in early, non-metastasized stages, but below 10%, when distant metastases have formed) and is responsible for more than 90% of cancer deaths. As exemplified for colorectal cancer (CRC), about 2 million cases are reported for now, which is prognosticated to be increased to 3 million in 2040, currently associated with a million of cancer deaths, which will be increased to more than 1.5 million in 2040. Therefore, it is still an important and urgent clinical need to better understand and to more successfully combat cancer metastasis.

To identify cancer patients at high risk for metastasis, biomarkers can be employed. Particularly those markers simultaneously acting as key drivers for metastasis are extremely desired as potential tools. Global translational concepts mainly aim at exploitation of novel key molecules in metastasis for prognosis and therapy of solid cancers. Clinical interventions targeting these molecules are of highest importance.

Cancer metastasis is a multistep process starting with dissemination of single cells or collective cell migration, intravasation and extravasation to the blood or lymphatic vessels, finally seeding at a distant organ site. A multitude of genes involved in metastasis have been identified in studies across numerous cancer types.

However, in search of new drivers of metastasis the gene MACC1, a novel, previously undescribed gene was identified in CRC samples. MACC1 induces fundamental processes like proliferation, dissemination, migration, invasiveness in cell culture as well as metastasis in xenografted and transgenic mice. MACC1 contributes to several important features of tumorigenesis, tumor progression and metastasis development, such as wound healing, colony formation, anti-apoptosis and inflammation, is decisively involved in stemness, circadian clock regulation, protein trafficking/clathrin-mediated endocytosis, biomechanics and, very importantly, chemoresistance.

In recent years, different regulations of MACC1 were reported: i.e. on the transcriptional, post-transcriptional, translational and post-translational level. Transcriptional targets and protein-protein interactors of MACC1 were unveiled as new diagnostic, prognostic and predictive key players for tumor progression and metastasis. By generating first transgenic MACC1 mouse models, MACC1-induced cancer transition and its link to stemness was discovered. Further, MACC1 networks – MACC1 together with regulating RNAs and/or proteins - were identified.

Following gene-specific MACC1 knockdowns/knockouts, newly identified small molecule inhibitors were employed, preventing MACC1-induced metastasis development in mouse models. Thus, the main focus is on cancer cell signaling cascades of metastasis-initiating genes/proteins and particularly of MACC1, their networks and axes, for improved prognosis and prediction of cancer patients in cross-entity studies (in addition to CRC) based on tumor tissue and patient blood and on intervention approaches for prevention/reduction of cancer metastasis employing repositioned drugs and novel compounds.

In this Research Topic, identification of the novel, dual function of GIPC1 is reported: it acts as protein interaction partner and as transcription factor of MACC1, involved in tumor progression and cancer metastasis (Siegel et al.). Endogenous, but not CMV promoter-driven MACC1 expression was lowered by GIPC1 knockdown, followed by diminished MACC1-induced *in vitro* phenotypes such as cell migration and invasion. In mice, intrasplenically transplanted with MACC1-overexpressing CRC cells, GIPC1 suppression reduced tumor growth and metastasis formation. Remarkably, in human primary CRC specimen, GIPC1 correlates with MACC1 expression and is of prognostic value for metastasis formation and metastasis-free survival. Thus, combining analysis of MACC1 and GIPC1 levels improved patient survival prognosis.

Further, Hohmann et al. summarized the current knowledge on MACC1-induced tumor cell motility, particularly cell migration. The authors specifically focus on cytoskeletal and adhesive systems. Several *in vitro* models employed for analyzing cell migration are summarized. Importantly, the authors point to issues with the currently most prevalent models used to study MACC1-dependent migration. Lastly, open questions about MACC1-dependent effects on tumor cell migration are addressed.

Triple negative breast cancer (TNBC) is a very aggressive form of breast cancer, for which clinical outcome is poor. Here, MACC1 was identified as one of the top candidate genes mediating the aggressive phenotype in the T1 tumor cells, unveiled by bioinformatic analysis and Kaplan Meier survival analysis (Thankamony et al.). The impact of MACC1 on the proliferative phenotype was validated and exploited for a therapeutic approach to target the T1 cell population using the small molecule lovastatin, a known transcriptional inhibitor of MACC1. This study adds to our understanding on the molecular basis of heterogeneity in breast cancer. In fact, this is decisive to improve the treatment of women who currently suffer from this highly aggressive TNBC subtype.

For nasopharyngeal cancer (NPC), the authors demonstrated that MACC1 and vimentin upregulation and E-cadherin downregulation were linked to reduced patient survival (Cheng et al.). Overexpression of MACC1 correlated with reduced 5-year overall, metastasis-free and disease-free survival. The univariate analyses showed, that the levels of the MACC1/E-cadherin/vimentin network in association with T/N tumor classification and cancer staging are prognostic factors for NPC. This supports the association of MACC1 and EMT in NPCs and strengthens the role of MACC1 as a prognostic biomarker and therapeutic target for treatment of this cancer entity.

The extent of MACC1 and MET protein expression was analyzed by immunohistochemical staining in a tissue microarray in cutaneous melanoma. This cancer represents the most lethal malignancy among skin cancers with a high metastatic potential (Zhou et al.). In metastatic melanomas, on average higher MACC1 expression was found compared to primary melanomas and nevi. However, MACC1 expression did not correlate with MET expression in nevi and primary melanomas. By contrast, this correlation was stronger in metastatic melanomas. Regarding other clinicopathologic factors including patient age, gender, histologic subtypes, depth of invasion, and staging, expressions of MACC1 and MET did not show differences. Therefore, this study concludes, that high MACC1 expression or expression of both, MACC1 and MET is associated with metastasis of cutaneous melanoma.

In this Research Topic, important and clinically relevant fields of MACC1-related research areas were addressed: epigenetic and transcriptional regulation of the MACC1 promoter activity, mechanisms of MACC1-induced phenotypes such as cell migration in tumors, MACC1 networks for improved patient prognosis, analyzed in different types of solid cancers. All of this new MACC1-based knowledge can be exploited (i) for improved diagnosis and prognosis and (ii) for response prediction of solid cancer patients by using their tumors and/or blood. Based on the MACC1 network in cancer, novel therapeutic approaches are currently being developed and tested in clinical trials. Patients with metastatic disease are treated using newly identified or repositioned small molecule inhibitors acting on this biomarker.

Taken together, MACC1 and the associated networks are valuable tools for cancer diagnosis, prognosis and prediction, and emerging targets for specific tailored approaches to intervene in metastasis formation. Thus, this Research Topic will draw more attention of readers from all fields of cancer research and clinical care to the importance of MACC1 networks and the great potential to combat metastatic disease for improved patient survival.

## Author contributions

WW: Writing – review & editing, Writing – original draft, Conceptualization. FS: Writing – review & editing, Writing – original draft, Conceptualization. US: Conceptualization, Writing – review & editing, Writing – original draft.
